# Selection of Nucleic Acid Aptamers Targeting Tumor Cell-Surface Protein Biomarkers

**DOI:** 10.3390/cancers9060069

**Published:** 2017-06-21

**Authors:** Marie-Cécile Mercier, Monique Dontenwill, Laurence Choulier

**Affiliations:** UMR 7213 CNRS, Laboratoire de Biophotonique et Pharmacologie, Tumoral Signaling and Therapeutic Targets, Université de Strasbourg, Faculté de Pharmacie, 67401 Illkirch, France; marie-cecile.mercier@unistra.fr (M.-C.M.); monique.dontenwill@unistra.fr (M.D.)

**Keywords:** aptamer, SELEX, cell-surface biomarker

## Abstract

Aptamers are nucleic acids referred to as chemical antibodies as they bind to their specific targets with high affinity and selectivity. They are selected via an iterative process known as ‘selective evolution of ligands by exponential enrichment’ (SELEX). Aptamers have been developed against numerous cancer targets and among them, many tumor cell-membrane protein biomarkers. The identification of aptamers targeting cell-surface proteins has mainly been performed by two different strategies: protein- and cell-based SELEX, when the targets used for selection were proteins and cells, respectively. This review aims to update the literature on aptamers targeting tumor cell surface protein biomarkers, highlighting potentials, pitfalls of protein- and cell-based selection processes and applications of such selected molecules. Aptamers as promising agents for diagnosis and therapeutic approaches in oncology are documented, as well as aptamers in clinical development.

## 1. Introduction

Nucleic acid aptamers are single stranded DNA (ssDNA) or RNA molecules. They are selected through a fully in vitro well-established iterative process known as ‘selective evolution of ligands by exponential enrichment’ (SELEX) [1,2,3]. A large library of 10^14^–10^15^ ssDNA or RNA sequences of 20–50 variable bases is combined with a target, under temperature and buffer conditions dependent on requirements. During the partitioning step, nucleic acid molecules which bind to the target are selected while the vast majority of sequences which do not bind are washed away. Binding molecules are amplified thanks to the fixed primer-binding sequences flanking the variable region, by PCR or RT-PCR according to the nature of the oligonucleotide, ssDNA or RNA. Double-stranded DNA molecules are then separated from ssDNA in DNA-SELEX, or in vitro transcribed in RNA-SELEX by means of a T7-RNA polymerase binding site in the fixed region. The three steps (selection, partitioning and amplification) constitute a round of SELEX. Negative selection to environmental elements (usually supports of the SELEX process, like filters, beads…) and/or counter selection to target’s counterparts (like related proteins or cells) is usually performed, either before or after the positive selection, to eliminate non-specific binding nucleic acids. The rounds of selection are repeated several times, increasing stringency progressively, until molecules with the desired binding properties are cloned and sequenced. Those selected oligonucleotides are named aptamers, from the greek aptus (“to fit”) and the suffix “-mer”. Databases dedicated to aptamers provide information on targets of specific aptamers, length, molecular weight, extinction coefficient, GC (guanine-cytosine) content, chemistry, primary sequences, predictive secondary structures, as well as affinities, assay buffers… (for example, [4,5,6]).

More than 900 aptamers have been generated from SELEX [7] towards a wide range of targets such as inorganic ions, small organic ligands, amino-acids, nucleotides and derivatives, oligonucleotides, antibiotics, peptides, proteins, sugars, parasites, virus, cells and tissues; the most common targets remaining proteins. In oncology, the aptamer field is rapidly expanding. On 3 May 2017, Medline listed 1398 entries for ‘aptamer and cancer’, including 167 reviews, with more than half entries published in the last 4 years. Deregulation of cell-surface membrane protein expressions and activities are hallmarks of cancer cells. Receptors that are uniquely expressed, overexpressed or mutated in tumors are major pharmaceutical targets for therapies in the era of personalized medicine. Advances in genomic, proteomic, metabolomic techniques led to the discovery of tumor biomarkers including cell surface receptors (tyrosine kinase receptors, cell adhesion receptors, cell death receptors) [8,9,10]. A vast majority of drugs are currently designed to target such proteins/receptors and to inhibit their oncogenic activities [11]. Recent data emphasized the high inter-and intra-tumoral heterogeneity of histologically similar tumors, at genomic but also protein levels of relevant targets [12,13]. Failure of clinical trials for targeted therapies in non-stratified patient populations may be linked to this underappreciated tumor heterogeneity. Highly specific tools, such as aptamers, may represent molecular probes for labeling the targets on tumor samples and thus helping to patient stratification. In addition, accessibility of cell surface receptors for targeting with aptamers will allow not only molecular imaging but also the direct delivery of drugs to relevant cancer cells [14].

The success of a SELEX process relates to the identification of aptamers of high affinities and specificities for their targets. Identification of aptamers is more complex for cell-surface proteins which activities depend on their conformation and on their cellular environment than for non-amphipatic molecules. To target cell surface biomarkers, it can therefore be important when possible to choose selection conditions and processes mimicking as far as possible the targets natural environment. This review will thus focus on the main selection strategies which have been used to identify aptamers to tumor cell-surface proteins, highlighting differences, success, and limitations of the SELEX processes. Tumor cell-surface biomarkers used as targets for SELEX are listed in Table 1. This review includes three more tables describing characteristics and applications of aptamers identified by protein- and cell-based SELEX, for which targets are proteins and cells, respectively. For cell-based SELEX, a distinction has been made between pre- and post-identified targets. Powerful potential applications of aptamers as diagnostic and therapeutic agents in the field of oncology will be briefly discussed and recent developments of the few aptamers already in clinical trials in oncology described.

## 2. Generalities on Aptamers

Owing to their three-dimensional conformation and the strong shape complementarity between aptamers and their targets, aptamers are known to recognize their specific target with high affinity and selectivity. Analogous to antibodies in their range of target recognition and variety of applications, they are referred as ‘chemical antibodies’ [15]. Aptamers though possess several advantages over antibodies: Temperature stability, self-refolding, easy production, and presumably lack of immunogenicity/toxicity… Due to their molecular weight, which is comprised between 10 and 30 Da, aptamers penetrate tissues faster and more efficiently than the 150 kDa antibodies [16]. Aptamers are chemically synthetized, rapidly, at reasonable cost, with no or low variation from batch to batch, and independently of any biological systems. Risks of viral and bacterial contamination are thus eliminated. Aptamers are thermally stable. Denatured at 95 °C, they refold into their correct three-dimensional conformation when cooled down to room temperature. To date, aptamers have never elicited toxicity in therapeutic applications.

RNA aptamers fold into more diverse three-dimensional structures than ssDNA aptamers, because of their 2′-hydroxyl (2′-OH) group on ribose and the non-Watson-Crick base pairing. The chemical production of ssDNA aptamers is easier and cheaper than RNA aptamers. But the preparation of ssDNA molecules during the SELEX process is more challenging than RNAs [17,18]. Nucleic aptamers suffer from two major disadvantages: their nuclease sensitivity, especially for RNA aptamers that can be cleared from the circulation within minutes or seconds [19], which limits their development as pharmaceuticals. Further, the chemical diversity of a 4 nucleotides based DNA and RNA library may be limited compared to that of a 20 amino acids based library. Developments allowed overcoming these weaknesses. The 2′-OH group is the naturally occurring cleavage site prone to nucleophilic attack. Typical modifications that can be inserted during the SELEX process include masking the nuclease-sensitive 2′OH group by replacing it with 2′fluoro (2′-F) or 2′-amino (2′-NH2). Such modified pyrimidines are incorporated via an efficient engineered mutant T7 RNA polymerase [20]. Other modified nucleotides can also be incorporated in SELEX libraries like 2′-O-methyl nucleotides, locked nucleic acid (LNA), hexitol nucleic acid (HNA) [21,22,23,24,25]. Modifications, if they do not alter much specificity and target binding properties of aptamers, can be introduced post-selection. Those modifications include 2′-F, 2′-NH2, 2′-thio (2′-SH), 2′-azido (2′-N3), 2′-hydroxymethyl (2′-CH_2_OH) or 2′-methoxy (2′-O-Me) groups, LNA [26]. Synthetic nucleic acids, named xeno nucleic acids (XNA), increase the resistance and also the diversity of such modified aptamers [27,28]. Gold et al. [29] demonstrated that the success rate of a conventional SELEX on difficult proteins is lower than 30% and improved this rate up to 84%, by the incorporation of four modified nucleotide triphosphate analogs: 5-benzylaminocarbonyl-dU(BndU), 5-naphthylmethylaminocarbonyl-dU (NapdU), 5- tryptaminocarbonyl-dU (TrpdU), and 5-isobutylaminocarbonyl-dU (iBudU). The idea was to expand the chemical diversity of aptamers by adding functional groups that mimic amino acid side chains [30]. These modifications enhance protein binding through direct hydrophobic contacts with the target protein, resulting in increased binding affinities and slower complex dissociation rates compared to unmodified aptamers. So called Slow Off-rate Modified Aptamers (SOMAmers), are developed by the SomaLogic Company (Boulder, CO, USA) to currently more than 1300 proteins. SOMAmers are used in SOMAscan^TM^ assay, a highly sensitive proteomic tool for discovering and validation of biomarkers. Capping aptamers with inverted nucleotides is another alternative to reduce exonuclease degradation [31]. The native phosphodiester backbone of nucleotides can also be modified with boranophosphate or phosphorothioates [32,33,34]. The company NOXXON Pharma AG (Berlin, Germany) takes advantage of spiegelmers (‘spiegel’ means ‘mirror’ in German) to prevent enzymatic degradation in biological fluids [19,35]. Spiegelmers are artificial RNA-like molecules built from L-ribose units, images of natural D-units, highly resistant to nuclease degradation and much more stable in vivo, with lifetimes greater than 60 hours in plasma. The SELEX is performed with D-RNA libraries combined with mirror images of peptides or small proteins artificially synthesized with D-aminoacids. Once aptamers are identified, they are converted to stable L-RNAs and they bind to the natural L-form of peptide or protein targets with equally high affinities. Three spiegelmers are actually in clinical trials, of which one in oncology (described in Section 5). This process is however limited to artificially synthesized targets. To increase their size, consequently reducing their systemic clearance and prolonging their in vivo half-lives, ssDNA and RNA aptamers are often coupled to bulky groups, such as poly (D,L-lactic-co-glycolic acid) (PLGA), poly ethylene glycol (PEG), liposomes, steptavidin or cholesterol [36,37,38,39,40,41].

## 3. Protein- and Cell-Based SELEX Processes

**Figure 1:**
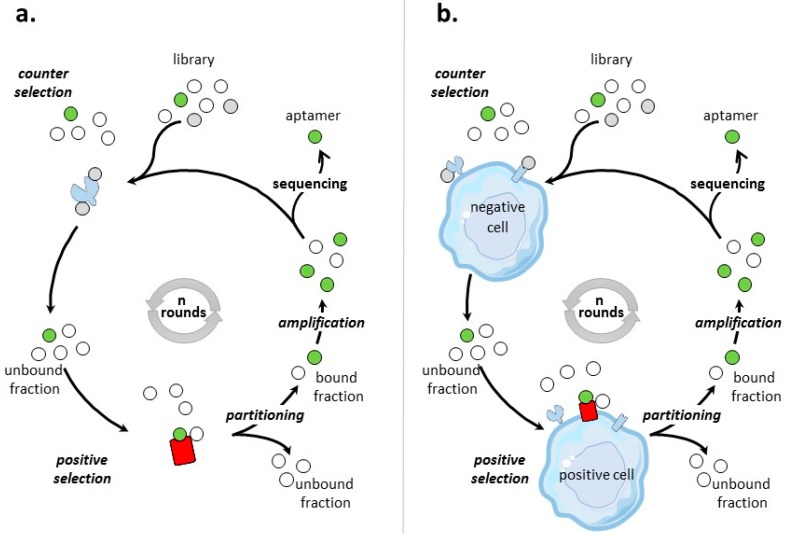

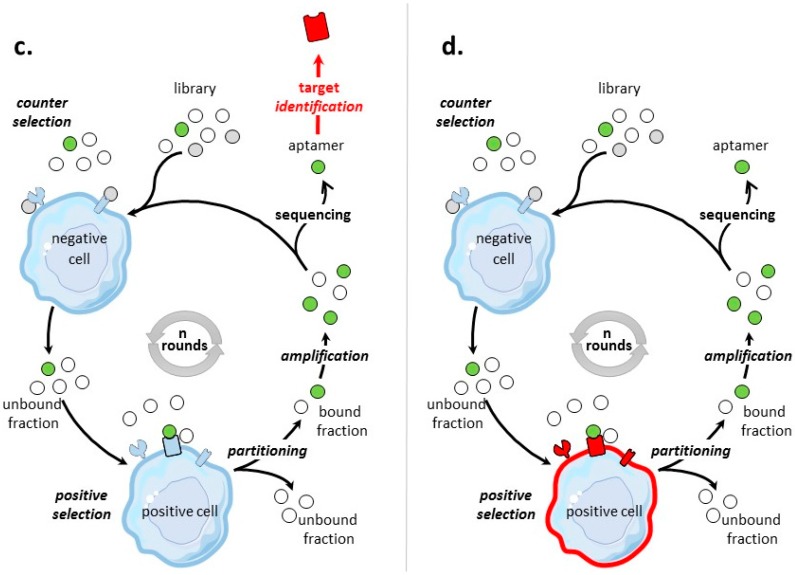
Scheme of protein- and cell-based SELEX processes. Briefly, the process of SELEX involves first a selection step: the nucleic acid library is incubated with a target (positive selection), which can be preceded or followed by a counter selection phase to remove non-specific nucleic acid molecules. During the partitioning step, bound and unbound fractions are separated. The bound fraction is amplified to obtain an enriched pool for next round of selection. This process is repeated for n rounds until the pool is enriched for sequences that specifically bind the target. These nucleic acid molecules are cloned and sequenced. Individual sequences are aptamers. (**a**) Protein-based SELEX. The pre-identified purified protein used as target for the protein-based SELEX is colored in red. (**b**) Cell-based SELEX on a pre-identified tumor cell-surface biomarker colored in red. (**c**) Cell-based SELEX on a post-identified tumor cell-surface biomarker. The target, identified at the end of the SELEX process, is colored in red. (**d**) Cell-based SELEX to a tumor cell type. In this case, no particular cell-surface biomarkers are identified and aptamers are specific to the cell’s molecular signature. The whole cell used for positive selection is colored in red.

Different SELEX processes have been developed for the selection of aptamers targeting tumor cell-surface protein biomarkers. Figure 1 illustrates the classical protein- and cell-based SELEX procedures, the two main methods that are used to identify aptamers to tumor cell-surface biomarkers for which targets are proteins and cells, respectively.

### 3.1. Aptamers Selected by Protein-Based SELEX

Protein SELEX is the simplest form of SELEX process (Figure 1a). Table 2 provides an inventory of most of tumor cell-surface protein biomarkers selected by protein-SELEX. Aptamers selected by protein-based SELEX have been reported for cell-adhesion molecules, tyrosine kinase receptors, cell membrane-associated enzymes, mucins, T-cell receptors, co-stimulator receptors, surface transmembrane glycoproteins… Some of the biomarkers have been subjects to different SELEX procedures, like integrin αvβ3, MUC-1, L-selectin and CD44 and the tyrosine kinase receptors EGFR, HER-2, and c-Met. Many factors varied, like the nature and the number of random nucleotides of the library, the separation procedures, the numbers of selection rounds. All of these selection procedures lead to aptamers of high affinities for their targets, usually in the low nanomolar range.

Cell surface proteins used as targets for protein-based SELEX are either full-length or truncated versions of full length proteins, generally recombinant ectodomains coupled to tags (His-tags, Fc fragments of antibody or GST), facilitating purification and selection by affinity. Peptides can also be used as targets. In that case, the advantages linked to the exact knowledge of the aptatope, and to the facility of production of large amounts of targets can be offset by the limitations of these peptides as protein mimics. Cell-surface proteins are amphipatic membrane proteins, and therefore not easily extracted from the lipidic membrane, purified and solubilized. Even though membrane proteins can be purified and solubilized, large amount of proteins are needed for a whole protein-SELEX procedure. Further, full-length membrane proteins or ectodomains, expressed in prokaryotic or lower eukaryotic systems, may lack or have different post-translational modifications (phosphorylation, glycosylation, ubiquitination, methylation, myristoylation, acetylation…). For example, the 2′F-RNA aptamer E21, elicited against the EGFRvIII ectodomain produced in bacteria, did not bind to the native protein expressed from eucaryotic cells because glycosylation, a post-translational modification present only in eukaryotic systems, significantly alters the structure of the target protein [58].

Some of the biomarkers have been subjects to different SELEX, like MUC-1, that allowed comparison between aptamers targeting proteins and peptides mimics of proteins. For example, Ferreira et al. [68] selected ssDNA aptamers targeting two peptides of the native MUC1. They used the 9-amino-acid immune-dominant antibody-binding epitope APDTRPAPG which contains only part of the variable tandem repeat region of MUC-1, as well as a larger peptide (60 amino acids long) which comprised three complete tandem repeats of the MUC1 region. From a preceding experience with an antibody binding to MUC1, the short peptide was weakly binding to the antibody compared to longer peptides, possibly due to increased flexibility resulting in higher entropic cost [86]. The two SELEX on the two peptides of different lengths resulted in identical aptamer sequences with affinities as low as 0.1 nM. Moreover, aptamers selected on the short peptide have been shown to successfully recognize and bind to the native MUC1 at the surface of the MCF7 breast cancer cell line. In comparison, the same group has selected aptamers to the MUC-1 protein with higher K_D_ (47–85 nM) than those obtained for aptamers to peptides, which might be due to an unstructured MUC-1 protein [69].

Overall, the main disadvantage of protein SELEX is that purified and solubilized membrane proteins, recombinant proteins and peptides may not adopt the same state (in terms of lengths, conformation, post-translational modifications…), than the cell-surface biomarker in its endogenous environment. Thus, some aptamers selected against purified membrane proteins failed to recognize their targets in whole cells [58,78], limiting their use for medical applications [87]. Moreover, some biomarkers like receptors require co-receptors for proper folding, that prevents the use of protein-SELEX. Other processes, like cell-based SELEX using whole living cells as targets may overcome these limitations.

### 3.2. Aptamers Selected by Cell-Based SELEX

The general process of cell-SELEX is shown in Figure 1b–d. In cell-based SELEX, the target of the selection is embedded in its native environment in cells. PubMed indicates 829 entries with ‘cell SELEX’ of which more than 1/3 are on ‘cancer and cell SELEX’. There has been growing developments in cell-SELEX these last years. Recent reviews detail advances in aptamer selection technology, particularly the high-throughput next-generation sequencing and bioinformatics [88], the cell-SELEX process [89], the evolution of complex target SELEX [90]. For example, FACS (Fluorescence activated cell sorting) SELEX aims at removing, from the cell suspension, dead cells which could have an impact on the selection [91,92,93]. In Internalized cell-SELEX, aptamers are selected on their ability to bind to cell-surface target and to internalize in the cell [94,95,96,97,98,99,100]. Nucleic acid sequences which do not internalize are eliminated. To best mimic the natural environment of targets, Souza et al. [101] developed 3D cell-SELEX, in which aptamers are selected on spheroid cells in 3D cell culture by magnetic levitation method to mimic the tissue microenvironment in vitro.

#### 3.2.1. Cell-Based SELEX to Identify Aptamers to Tumor Cell-Surface Protein Biomarkers

Depending on the objective, knowledge of the target identity prior to selection can be useful, or not. Therefore, different strategies can be distinguished:

##### Pre-Identified Tumor Cell-Surface Biomarkers

When the objective of a cell-based SELEX is the identification of aptamers to a clearly identified cell-surface biomarker (Figure 1b), the SELEX procedure is sometimes termed TECS-SELEX for ‘target expressed on cell surface-SELEX’ [102]. In Table 3, selections by cell-based SELEX realized on pre-identified tumor cell-surface biomarkers are described. Many identical tumor biomarkers, in particular tyrosine kinase receptors, EpCAM and integrins, were used as pre-identified targets for protein-and cell-based SELEX (Table 2 and Table 3). As an example of solid tumor targeted by cell-based SELEX, Delac et al. [103] reviewed aptamers designed to target glioblastoma cells. Some of the key cell surface molecules that have been identified as glioblastoma biomarkers (transferrin receptor, αvβ3 integrin, EGFR, EGFRvIII) [104], have already been targets of protein-and/or cell-based SELEX (Table 2 and Table 3).

Different procedures have been developed to direct the recognition of the target by the aptamer. For example, ligand-guided selection (LIGS) SELEX guides the selection to a specific epitope of a known cell-surface target. Zumrut et al. [105,106] used antibodies as secondary ligands to elute specific aptamers. The antibody interacts with its cognate antigen to outcompete aptamers specific for a pre-determined cell-surface receptor and replace specific aptamers from an enriched SELEX pool. Immunoprecipitation (IP) SELEX is an approach combining cell-SELEX and protein-nucleic acid immunoprecipitation to ensure that aptamers bind to a pre-identified target [7,107]. Hybrid-SELEX, also called cross-over SELEX, is a combinatorial approach to guide selection towards a pre-identified target. Usually, the first rounds of selection are realized on cells which express the target by cell-SELEX, and then rounds of selection are realized on the same version of the target in its purified form by protein-based SELEX. A reverse hybrid-SELEX combines first protein-SELEX followed by cell-SELEX. Hybrid-or reverse hybrid-SELEX have been used to isolate aptamers targeting the RET^C634Y^ mutant receptor tyrosine kinase overexpressed in PC12 cells [108], the HER-2 tyrosine kinase receptor expressed in SKOV3 ovarian cancer cells [109], CD16α on Jurkat cells overexpressing CD16 alloforms [64], integrin α6β4 on PC-3 cells [110], the transferrin receptor CD71 expressed in HeLa cells [94]. In this last example, a combination of protein-SELEX and cell-internalized SELEX was realised. Soldevilla et al. [111] used another variant of reverse hybrid-SELEX, combining cell-SELEX to peptide-SELEX. Ten rounds of selection were first realized by peptide-SELEX, followed by one round of cell-SELEX on a chemotherapy-resistant tumor cell line that highly express MRP1 (H69AR).

Cell-based SELEX involves positive selection to collect aptamers that interact specifically with the target cells and usually counter selection to eliminate nonspecific nucleic acid sequences binding to negative cells. Different human tumor cell lines have been used for positive selection: glioblastoma (U251, U87MG), breast carcinoma (SKBR-3, N202.1), ovarian cancer (SKOV3), gastric cancer (N87), lung carcinoma (A549, H69), lymphoma (Jurkat, K299T), liver cancer (HepG2), prostate adenocarcinoma (PC-3), cervical cancer (HeLa). The PC12 cell line derived from pheochromocytoma of the rat adrenal medulla has also been used, as well as other non-tumoral cells lines, like Chinese hamster ovary (CHO), Human embryonic kidney (HEK) cells. The choice of the couple of cells used for positive- and counter-selections is of great importance for cell-based SELEX on pre-identified cell-surface protein. The two cell type should display a high difference in target expression levels, so that aptamers interact specifically with the target on positive cells and does not interact with cells used for counter-selection which does not display or displays lower levels of the target protein. For positive selection, the pre-identified target can be naturally expressed or over-expressed at the surface of the cell line. Cells used for counter selection are chosen in light of positive cells. In the literature, cell lines used for counter selection were:

(i) isogenic to cells used for positive selection. Most of the cell-based SELEX process used cells overexpressing the target of interest for the positive selection, and the parental cell line for counter selection (Table 3). As an example over many others, a cell-based SELEX was realized on Chinese hamster ovary (CHO)-K1 cells ectopically expressing human transforming growth factor-β type III receptor while CHO-K1 cells were used for counter selection [102]. In two examples described in Table 3, both on integrins, cells used for counter-selection were under-expressing the target. In the first example, the parental isogenic cell lines are used for the positive selection. Berg et al. [110] used this strategy to select an ssDNA aptamer specific for α6β4 integrin in its native state. Five rounds of positive cell selection were realized on PC-3 cells. PC-3 β4 integrin (ITGB4) knockdown cells were used in a preselection step to deplete aptamers specific for cell surface marker other than β4 sub-unit. However, the counter-selection strategy was found to be insufficient. And seven more rounds of protein-SELEX were performed on a recombinant α6β4 protein to select for α6β4-specific aptamers. In the second example, HEK293 cells lines are used. For positive selection, HEK293 cells were manipulated to generate positive αv selection cells by overexpressing ITGAV. For counter selection, the same cell line was depleted in ITGAV with microRNA-mediated silencing. Takahashi et al. [112] named this strategy Icell-SELEX for isogenic cell SELEX.

(ii) unrelated to cells used for positive selection. Two studies [113,114] related the use for positive selection of the breast cancer cell line SK-BR3 modified to overexpress HER-2. For counter-selection, the human breast cell types MDA-MB-231 and MDA-MB-468, respectively underexpressing and negative for HER-2 were used. Twelve and sixteen rounds of selection were performed to select RNA- [113] and ssDNA- [114] aptamers. According to Dastjerdi et al. [114], the use of the HER2 underexpressing cell line (MDA-MB-231) in Kang et al’s study [113], instead of a HER2-negative cell line may have compromised counter selection. A hybrid-SELEX strategy was used to get aptamers to the surface transmembrane glycoprotein CD30 [115]. For the cell-SELEX, lymphoma cells K299T and Jurkat cells were used for positive and counter-selection, respectively.

(iv) inexistent or not documented [116]. ssDNA aptamers to HER-2 have recently been selected through a hybrid-SELEX [109], combining 8 rounds a protein-SELEX using the His-tagged extracellular domain of HER-2 and 7 rounds of cell-SELEX on HER-2 over-expressed in SKOV3 cancer cells. Human serum albumin was used for negative screening in protein-SELEX and added to ssDNA pools during cell-SELEX, but no cells were used for counter-selection. Post-selection, HER2-negative MCF7, MDA-MB-435 and MDA-MB-231 cells were used to test the specificity of aptamers. Seven aptamer candidates from hybrid-SELEX were radiolabeled with ^18^F and further screened in vivo by PET imaging in a SKOV3 tumor model. Two aptamers, Heraptamer-1 and-2 which had relatively high tumor uptake ratios, are promising ligands for HER-2 imaging in cancer. A similar approach was used by Wilner et al. [94]. Four rounds of protein-SELEX were performed using the His-tagged recombinant transferring receptor CD71 (TfR) as target, followed by 1 round of cell-SELEX on HeLa cells, a human cervical cancer cell line known to express TfR. Following the first round, a negative selection step was performed in which the library was pre-incubated with Ni-NTA agarose prior to the positive selection. But the cell-SELEX procedure did not include counter-selection steps.

##### Post-Identified Tumor Cell-Surface Biomarkers—Discovery of Tumor Biomarkers

Aptamers are valuable tools to isolate tumor-specific biomarkers [122,123,124], as cell-based SELEX can also be used to identify targets after the selection (Figure 1c). The idea is first to select and identify aptamers specific for a tumor cell type, and then to use these high-affinity aptamers as probes to identify their cell-surface targets. These cell-surface targets, specific for tumor cells under study, are identified as (new) biomarkers. The main challenge is the identification of the protein target, usually by affinity purification followed by mass-spectrometry. The first post cell-SELEX identification of a tumor biomarker was realized in 2003 by Daniels et al. [125]. The U251 glioblastoma cell line was used as target for positive selection. Counter selection rounds were not included in SELEX. Twenty-one rounds of cell-SELEX using a 34 nucleotide long random library of ssDNA were performed to select aptamer GBI-10, shown to be the ligand for Tenascin-C, which is not a cell-surface receptor, but an extracellular matrix protein. Table 4 presents some (certainly not all) tumor cell-surface protein biomarkers which have been identified post-SELEX.

The ssDNA aptamer AS1411 in clinical trials as a novel treatment for cancer is original compared to all other aptamers cited in this review, as its discovery does not result from a SELEX approach. It is referred as an aptamer as its activity arises from binding to a specific target via shape-specific recognition. Briefly, Bates et al. [84], in the late 1990’s, tested G-rich oligonucleotides (GRO) in a variety of cell lines and showed that they had unexpected anti-proliferative effect. AS1411 is a 26-nucleotide GRO, which forms quadruplex. It blocks the activation of secondary targets (NF-κB, and B-cell lymphoma 2, BCL2), sensitizes tumor cells to chemotherapy, has direct anti-proliferative effects in vitro, is efficiently internalized even at nanomolar doses… [126,127]. Later, aptamer AS1411 was found to target nucleolin [127,128], a multifunctional protein that was first considered to be nucleolar. Nucleolin was found to be highly expressed by cancer cells, intracellularly but also at the cell surface. A recent review described uses and mechanisms of the G-quadruplex oligonucleotide AS1411 as a targeting agent [129].

Berezovski et al. [139] described a technology for biomarker discovery, named aptamer-facilitated biomarker discovery (AptaBiD). It involves working with pool of aptamers rather than individual sequences. It is based on three steps: (i) cell-SELEX is performed to select ssDNA aptamers to biomarkers differentially expressed at the cell surfaces; (ii) a pool of biotinylated aptamers is used to isolate biomarkers from the cells, and then (iii) biomarkers are identified. AptaBiD was used to discover previously unknown surface biomarkers that distinguish live mature and immature dendritic cells [139] and to select DNA aptamers on lung adenocarcinoma cells derived from post-operative tissues [140].

##### Undetermined Targets

By cell-based SELEX process, aptamers have also been selected for tumor cell types (Figure 1d), without pre- or post-identification of targets. In this respect, aptamers are specific to the molecular signature of a tumor cell type. Such cell-based SELEX have been performed on several tumor cells, including breast cancer cells [141], colorectal cancer cells [142], small-cell lung cancer [143,144], non-small cell lung cancer [145], liver cancer cells [146], leukemia cells [147,148], gastric cancer cells [149], pancreatic ductal adenocarcinoma cells [150], prostate cancer cells [101], metastatic cells [151,152], and tumor initiating cells [153]. For example, J.N. Rich’s team [153], identified tumor initiating cell (TIC) specific ssDNA aptamers. They used a cell-based SELEX approach with positive selection for CD133(+) TICs and counter selection for CD133(-) non-TICs both from human GBM xenografts in mice. In addition, counter selection was also made on human non-tumoral neural progenitor cells. Selected aptamers specifically bound to TICs with dissociation constants (K_D_) in the low nanomolar range. These aptamers internalized into glioblastoma TICs that self-renew, proliferate, and initiate tumors.

#### 3.2.2. Advantages and Limitations of Cell-Based SELEX

Aptamer binding is dependent on the target conformation which is conditioned by its environment. Full-length purified recombinant proteins, ectodomains or peptidic fragments of proteins used in protein-based SELEX may not be relevant targets if they have to be used in clinical developments. In cell-based SELEX, cell-surface targets post-translationally processed are in their endogenous environment, as close as possible to their natural distributions and conformations. Cell-based SELEX does not necessitate the production and purification steps usually required for protein-based SELEX and therefore the method may be useful to target proteins difficult to be produced and/or purified. Moreover, aptamers can be selected without prior knowledge of cell-surface targets. In that case, the aptamer acts as a bait to identify its target, which can be a new tumor protein biomarker.

But cell-based SELEX is a complex process, much more difficult than protein-based SELEX [122]. Cells lines need to be available, cultivable and stable. Aptamers might be difficult to generate for less expressed target proteins on cells. For most of the tumor cell-based selections described in this review, SELEX required modifications of cell lines, like over- and/or under-expression of the cell-surface protein target for selection and/or counter-selection. Over- or under-expression of a protein might deregulate expression of other proteins. This can trigger modifications to the cell line that might not represent anymore the disease cellular context. It is commonly admitted that more rounds of positive selection are performed in cell-SELEX compared to protein-SELEX and more rounds of counter selection are required to improve the selectivity of aptamers.

Post-SELEX characterization of the target is often required to check if the predetermined target is the ‘real’ target of selected aptamers, or to identify the target if it has not been identified prior selection. Selected aptamers are used as bait, a posteriori, to purify and then to identify their targets. Briefly, biotinylated-aptamers are used to retain whole live cells on streptavidin beads. The cell lysate is then loaded on a SDS-PAGE gel. After electrophoresis and silver staining, proteins that have a higher intensity than controls (extracted proteins with empty beads and extracted proteins with a scrambled nucleic acid sequence) are extracted from the gel and analyzed by nano-LC-MS/MS mass spectroscopy [132,154]. Aptamer-mediated target identification is not an easy process, due to the inherent properties of lipid embedded proteins (hydrophobicity, poor solubility, resistance to digestion…), even though significant advances have occurred to address these challenges [155].

Other methods, indirect compared to the method described above, have been used to identify a protein target post-cell-based SELEX. For example, Esposito et al. [130] used different techniques to characterize an aptamer’s target. They realized a whole-cell-based SELEX strategy on human NSCLC to select 2′-fluoro RNA aptamers, which distinguish A549 cells (used for positive selection) from H460 cells (used for counter selection). The best candidate, CL4, was used to identify functional targets using a phospho-RTK array analysis, which provided convincing evidence that the target could likely be EGFR and/or ErbB3. To definitively identify the target, authors performed first a filter binding analysis with the soluble extracellular domains of EGFR and ErbB3, which showed a strong affinity for the EGFR protein (in its monomeric and dimeric forms), while no binding was observed for the ErbB3 protein. Binding of the radiolabeled CL4 aptamer to EGFR was confirmed on NIH3T3 cells overexpressing EGFR and compared to NIH3T3 cells. Binding to A549 cells was decreased by interfering with EGFR expression and by high concentration of EGF. Binding of radiolabeled CL4 on A549 cells was competed by soluble EGFR but not by soluble ErbB3. The specific interaction of CL4 with EGFR on cell surface was further analyzed by affinity purification on streptavidin coated beads of extracts from A549 cells treated with biotin-labeled CL4 followed by immunoblotting with anti-EGFR antibodies. Then, CL4 was detected on stable tumor derived cell lines that express high levels of EGFR. Later [156] this aptamer has been shown to bind to the EGFRvIII mutant, even though the mutant lacks most of domains I and III in the extracellular part of the protein. Other methods have been used to demonstrate the binding of CL4 to glioblastoma U87MG cells stably transfected with the EGFRvIII mutant: reverse transcription polymerase chain reaction, confocal microscopy with fluorescent FAM-labelled aptamer and flow cytometry. The binding was shown to inhibit EGFRvIII autophosphorylation and to affect migration, invasion and proliferation of glioblastoma cells.

Cell-based SELEX might lead to the selection of aptamers against other proteins than the pre-identified target. A cell-based SELEX was performed by Cibiel et al. [157] to identify aptamers against CHO-K1 cells expressing the Endothelin type B receptor (ET_B_R), using the isogenic CHO-K1 cell line for counter selection rounds. None of the selected aptamers could discriminate between both cell lines. Nevertheless, the authors decided to study the 2’-fluoro RNA aptamer ACE4. The band excised from the PAGE-gel revealed that ACE4 did not interact with ET_B_R, but with Annexin-2, a calcium dependent phospholipid-binding protein which is up-regulated in various tumor types and plays multiple roles in regulating cellular functions (angiogenesis, proliferation, cell migration and adhesion). The high affinity aptamer, although not initially planned to bind to the post-SELEX identified tumor biomarker Annexin A2, could however be a promising tool for biomedical applications. In another example [158], a panel of G-rich ssDNA aptamers was selected by cell-SELEX against EGFR-transfected A549 cell line, using A549 cells for counter selection, and their targets was suggested to be nucleolin.

## 4. Applications of Aptamers in Oncology

Besides their role for biomarker discovery above mentioned, aptamers have many applications in oncology. Some relevant applications of aptamers in the cancer field will be briefly described.

### 4.1. Aptamers as Detection and Imaging Reagent

Current approaches of protein biomarker detection include gel electrophoresis and conventional immunoassays (for capture and/or detection) such as enzyme-linked immunosorbent assay (ELISA), surface plasmon resonance, mass-sensing BioCD protein array, surface enhanced Raman spectroscopy (SERS), microfluidics and colorimetric, electrochemical and fluorescence assays (flow cytometry, europium or gold nanoparticle based detection, protein microarray, quantum dots…). Most of these approaches still lack accuracy, sensitivity and specificity required for diagnostic application. Advantages and limitations of these methods have been compared for the prostate specific antigen (PSA) biomarker detection [159]. Different cancer biosensors and their characteristics were recently reported [160]. Aptamers are a class of bio-recognition element which can be used as molecular tools for clinical diagnosis [161,162]. For localization of proteins on tissue sections to aid in histopathological diagnosis, aptahistochemistry methods have been developed [163]. Soluble tumor biomarkers (notably CEA, EGFR, HER2, MUC1, PSA) were detected on aptamer-based analytical platforms with low limits of detection [164]. Aptamers can be used in sufficiently sensitive diagnosis assays for the detection and capture of circulating tumor cells [165,166]. Upon the binding to their targets, conformational changes undergone by aptamers can be converted into measurable signals. So called aptasensors match the requirements of fast and portable molecular devices. The most current applications of aptasensors in oncology concerns biomarker detection [167]. A recent review reports the development of aptasensors for analysis of tumor biomarkers using electrochemical, electrochemoluminescence and photoelectrochemical transduction. In particular, nano-structure-based aptasensors significantly improved analytical performances [168]. Aptasensors can also be used to detect exoxomes [169]. Aptamers can be used as imaging agents [170,171,172,173], for example to track cell status and functions [174]. In particular, glioblastoma-specific aptamers were developed by either targeting the whole cell surface or known glioma biomarkers. Coupled with radionuclide or fluorescent labels, bioconjugates or nanoparticles, they represent a noninvasive manner for defining the tumor tissue border [103].

### 4.2. Aptamers in Therapy

Applications of aptamers as targeted therapeutics have been reviewed recently [15,175,176]. Nucleic acid aptamers can serve as antagonist or agonist and have therefore a direct therapeutic effect by themselves by activating or blocking key cellular functions upon interaction with their cellular targets. Particularly, aptamers have been successfully used as agonists of co-stimulatory receptors, like CD28, OX40 and 4-1BB, in aptamer-mediated cancer immunotherapy [177,178,179]. Multivalent aptamers which induce receptor multimerization, are often more efficient than monovalent aptamers to trigger downstream signaling [87]. Aptamers can also be used as delivery tools of therapeutic agents [15,180,181,182,183]. Internalized receptors carry along loaded aptamers [184]. Aptamers are thus cargoes which deliver therapeutics inside the cytosol of specific targeted cells where therapeutics exert their intracellular actions. Aptamer chimera, composed of an aptamer and a therapeutic nucleic acid, may target cancer cells and deliver anti-cancer nucleic acids, e.g. small interfering RNA, micro RNA, antimicroRNA and small hairpin RNA, for aptamer-mediated gene therapy [185]. Aptamers can also act as carriers for anti-tumor drugs or toxins [186,187]. Therapeutic agents are either non-covalently or covalently conjugated to aptamers. Doxorubicin, a chemotherapeutic agent extensively used in the treatment of various cancers, has often been used as a drug non-covalently conjugated to aptamers containing CG/GC sequences or covalently conjugated to aptamer through a functional linker [87]. Other aptamer-drug or-toxin conjugates include gemcitabine, docetaxel, daunorubicin, cisplatin, gelonin. For aptamer-mediated therapeutics delivery, aptamers have also been associated to nanoparticles [87,188,189,190,191,192,193]. Such aptamer nanocomplexes can be composed of copolymers, liposomes, metal nanomaterials (like gold nanoparticles, [194]), or virus-like particles.

## 5. Aptamers in Clinical Trials

Eleven aptamers have entered clinical stages [175,195] for eye disorders (three aptamers), coagulation (four aptamers), inflammation (two aptamers) and cancer (two aptamers). The majority of aptamer targets in clinical trials are extracellular proteins. The advantage linked to an extracellular protein is that the aptamer activity can be reversed by the use of an oligonucleotide antidote. Pegnivacogin is an antithrombotic aptamer that binds and inhibits coagulation factor IXa. The pegnivacogin dose-dependent antithrombotic activity can be reversed with an antidote, anivamersen, a 15-nucleotide long 2′-*O*-methyl RNA [196,197]. Pegnivacogin is a 2′-fluoro-2′-*O*-methyl 31 nucleotide long RNA aptamer, protected from exonuclease degradation by a 3′ inverted deoxythymidine cap. It is branched with a 40 kDa PEG carrier. PEGylation is generally used to increase aptamers molecular weight, prolong their circulating half-life by limiting renal clearance, enhance their stability and decrease their toxic accumulation in tissues and the volume of distribution of therapeutic molecules [198,199,200]. Tested in a phase IIb study, in patients with acute coronary syndrome, pegnivacogin administration resulted in allergic reactions after the first dose in three patients of 640. Two of them presented an anaphylactic reaction and one presented an isolated dermal reaction. These observations induced an early termination of the trial [201]. A phase III clinical trial (NCT01848106), enrolling 3232 patients undergoing percutaneous coronary intervention, terminated at an early stage, due to severe allergic reactions reported in 1% of patients receiving pegnivacogin versus <1% of patients treated with bivalirudin [202,203,204]. However, it was observed that pegnivacogin did not induce inflammation response or histamine release. This aptamer, or its degradation, was therefore not responsible for the observed severe allergic reactions. Patients with allergic reactions had high levels of antibody to PEG, induced by complement activation and trypase release [205]. In 1983 was first reported the induction of antibodies against PEG in animals [206], but induction of PEG immunogenicity became clearer these last years as more PEGylated therapeutics enter clinical trials [207,208,209]. Currently formulated with a 40 kDa PEG carrier, pegnivacogin is associated with severe allergic reactions [203].

Pegaptanib sodium or Macugen [210] was the first aptamer-based drug approved by the U.S. Food and Drug Administration, in December 2004. Pegaptanib sodium is a 2′-fluoropyrimidine RNA-based aptamer, 28 nucleotides in length that terminates in a pentylamino linker, to which two 20-kiloDalton monomethoxy PEG units are covalently attached. It recognizes the abundant isoform of vascular endothelial growth factor, VEGF_165_. As an inhibitor of VEGF_165_-associated vessel formation, pegaptanib sodium is an anti-angiogenic medicine for the treatment of wet age-related macular degeneration. Pegaptanib sodium has also potential therapeutic effects as an agent for solid cancers characterized by extensive angiogenesis [15].

In oncology, two aptamers have undergone clinical trials. The first one was AS1411 (AGRO100), a DNA quadruplex targeting nucleolin. This multifunctional protein, located in the nucleolus but overexpressed at the membrane of several cancer cells, is implicated in cancer development by interacting with key oncogenes (bcl-2, Rb, p53, Akt-1) and by transferring specific extracellular ligands into the cells. The interaction of AS1411 and nucleolin induces internalization, inhibition of DNA synthesis and induction of apoptosis [127,211,212,213,214]. In a phase I study (September 2003 and July 2004), AS1411was tested in advanced cancer. Results showed that 8/16 patients had a stable disease during 2–9 months and one patient presented a complete response after more than 6 months [215]. In another phase I study (NCT00881244, September 2003–April 2009) [216], AS1411 was tested in patients with advanced solid tumors. AS1411 was well-tolerated. It induced a partial and a complete response in twelve patients with metastatic renal-cell carcinoma (RCC) and a stabilization of disease (>2 months) for seven patients. In 2008, Aptamer AS1411 was tested in a phase II study, in monotherapy, in patients with advanced metastatic RCC who had failed treatment with >1prior tyrosine kinase inhibitor (NCT00740441, August 2008–September 2009). AS1411 has been administrated at 40 mg/kg/j for the 4 first days of a 28 days cycle for two cycles. Out of 35 patients, 33 finished the two cycles. One patient presented a partial response. The OOR was only 2.9% (24 months). Twelve (34.3%) patients presented a stabilization of disease (5.5 month) and 21 patients (60%) presented a progression of disease. Adverse effects were observed on 12 grade 1 or 2 patients. This study showed a low level of activity of AS1411 in patients with metastatic RRC. An important and durable response has however been observed without toxicity [217]. AS1411 has also been evaluated in a phase II study combined with cytarabine (chemotherapy) for the treatment of patients with primary refractory or relapsed acute myeloid leukemia (NCT00512083, December 2009–February 2011). Few patients presented a durable remission. But the drug combination was proved to be safe (40 mg/kg/day for 7 days), well tolerated and shows promising signs of activity [218].

NOX-A12 (olaptesed pegol) is in clinical development for the treatment of chronic lymphatic leukemia and multiple myeloma [219,220,221]. NOX-A12 is a 45 nucleotide long L-stereoisomer RNA aptamer (Spiegelmer®, NOXXON Pharma AG, Berlin, Germany), which is a specific antagonist of CXCL12/SDF-1 (C-X-C motif ligand 12/stromal cell-derived factor 1). This chemokine, via interaction with the receptors CXCR4 and CXCR7, induces attraction and activation of immune and non-immune cells, migration and adhesion of tumor cells to the protective tissue microenvironment. In tumor cells expressing a high level of CXCR4, the disruption of the CXCR4-CXCL2 interaction induces a diminution of the interactions of cancer cells with the bone marrow environment. This cell mobilization sensitizes cancer cells to therapy. NOX-A12 has been evaluated in two phase I and two phase II clinical studies. In a phase IIa study, in combination with bendamustine and rituximab (chemotherapy) in patients with relapsed/refractory chronic lymphocytic leukemia (CCL) (NCT01486797 march 2012 and January 2016) [222], results showed an effective long-term mobilization of CLL cell and an increased CLL cells circulating in the periphery blood. In term of clinical efficacy, all patients responded to the combination of chemotherapy and NOX-A12. The ORR was 100%. A partial response was achieved for 19/28 patients (68%) and a complete remission for 4/28 patient (14%). Among them, 8 high-risk patients, who had relapsed within 24 months after the first fludarabine/bendamustine treatment or presenting TP53-deletion/mutation, presented a partial or complete response. NOX-A12 was safe and well tolerated. In another independent phase IIa clinical study, NOX-A12 has been evaluated in combination with a proteasome inhibitor, bortezomib, and a corticosteroid, dexamethasone (VD) in previously treated patients with multiple myeloma. This study started in March 2012 and finished in October 2015 (NCT01521533) [223]. A mobilization of myeloma cells was shown in plasma. The OOR was estimated at 68%. Five patients (18%) presented a very good partial response, 2/28 (7%) a complete response and 12/28 (43%) a partial response. These studies showed that aptamer NOX-A12 merits further study in randomized controlled trials.

## 6. Conclusions

Due to their pleiotropic characteristics and improvements in the SELEX technology, aptamers, which can be selected to virtually any type of targets, are thought to gain rapidly an important place in clinical applications and may replace in many cases animal-derived antibodies. Aptamers as synthetic molecules are believed to save cost and time in R&D and manufacture. The targets of aptamers actually in clinical trials [175] are extracellular proteins (VEGF165, C5, PDGFβ, coagulation factor IXa, A1 domain of von Willebrand factor, thrombin, TFPI, CXCL12, CCL2, hepcidin peptide hormone), except nucleolin which is highly expressed at the surface of several tumor cells. Most of the aptamers in clinical trials were identified by protein-SELEX. Combination of protein-and cell-based selection processes may allow the discovery of highly selective but also conformation-dependent aptamers against tumor cell membrane biomarkers. Other selection processes more difficult to settle than protein-and cell-based SELEX and for which targets cannot be determined before selection, have been described to target a tumor under near-in vivo conditions. Aptamers have been selected on tissue sections [224,225], and very recently by Morph-X-Select (morphology based tissue aptamer selection, a combination of image-directed laser micro-dissection technology with tissue SELEX) on targeted tissue sections for ovarian cancer biomarker discovery [226]. Such selected DNA thio-aptamers have shown specificity to tumor vasculature of human ovarian tissue and human microvascular endothelial cells but not to the tumor stromal cells. Aptamers selected in living animals, through in vivo tissue-specific SELEX, have been shown to localize to hepatic colon cancer metastase [227] or to enter the brain after peripheral delivery [228]. These processes should prove helpful to characterize new tumor cell surface protein biomarkers, post-selection. Exciting new fields for aptamer applications in oncology emerge nowadays including detection of circulating tumor cells or tumor exosomes in biological fluid, and specific delivery of therapeutics inside cells with possibilities of co-targeting multiple oncogenic pathways. New opportunities for aptamers as multipotent tools will be characterized and will provide the pavement for approved clinical applications in the near future.

## Figures and Tables

**Table 1:** Major functions of biomarkers cited in this review.

Biomarker	Major Functions of Biomarker in Cancer
**Cell-adhesion molecules**	
Epithelial cell adhesion molecule-EpCAM (CD326)	EpCam is a pleiotropic molecule able to promote and prevent epithelial cell-cell adhesion. Induces proliferation, up-regulates proto-oncogene (c-myc, cyclins A/E) and is involved in migration, proliferation, differentiation and metastasis. Circulating tumor cell marker Implicated in self-renewal of stem cells
Carcinoembryonic antigen-CEA (CD66e)	Implicated in cell adhesion, anoikis resistance, promotion of metastasis
Integrin αvβ3	Essential for endothelial cell survival. Involved in cell growth and migration, tumor invasion, metastasis and angiogenesis
Integrin α6β4	Implicated in tumor cell growth, invasion and metastasis
Integrin αv	Endothelial adhesion receptor. Implicated in angiogenesis
Integrin α4	Prognostic marker in lymphocytic leukemia
E-and P-Selectin	Implicated in tumor cell adhesion to vascular endothelium, migration, proliferation of cancer cells and step of metastasis formation
L-Selectin	Roles in cell adhesion and in initiation of cell-cell interaction in vasculature system. Initiates events leading to leukocyte extravasations for the blood. Biomarker of naïve T cells
**Tyrosine kinase receptors**	
EGFR	EGFR is a receptor tyrosine kinase expressed at the surface of cell. Involved in proliferation, invasion, angiogenesis and metastasis. Associated with poor prognosis
EGFRVIII	The most common form of EGFR mutation. Associated with poor survival of patients. Promotes tumor cell motility
PDGFR β	Cell surface tyrosine kinase receptor. Implicated in proliferation, migration and angiogenesis
HER-2	Belongs to ErbB family (EGFR, ErbB2). Implicated in formation, progression of human tumor, aggressiveness and resistance to therapy. Implicated in poor prognosis
HER-3	Member of the receptor tyrosine kinase. Contributes to increased drug resistance in HER2 cells
c-MET	Receptor tyrosine kinase involved in development and progression of cancer. Induces cell proliferation, cell survival, cell motility and invasion
Receptor tyrosine kinase-RET^C634Y^	Mutated form of RET (proto-oncogene)
Neurotrophin receptor TrkB	Cancer cell resistance to chemotherapy. Promotes growth of cancer cells. Implicated in cell proliferation, differentiation, survival, and invasion
TGFβ III receptor	Role in signal transduction. Role in disease is poorly understood but correlates with higher tumor grade
Protein Tyrosine Kinase 7-PTK7	Biomarker for leukemia
Insulin Receptor	Receptor tyrosine kinase. Implicated in activation of MAPK/ERK and PI3K/AKT pathways Implicated in cancer development and progression
Axl	Belongs to TAM family of tyrosine kinase receptors. Implicated in invasiveness and metastasis
**Cell membrane-associated enzyme**	
Prostate specific membrane antigen-PSMA	Prostate tumor cell marker
**Mucines**	
MUC-1	Cell surface mucin glycoprotein
**Tumor necrosis factor receptor (TNF-R) and co-stimulatory receptors**	
T-cell receptor OX40	Member of TNF family receptors. Co-stimulatory receptor on CD4+ T cells. Increases immune response to cancer and foreign entities. Implicated in exacerbating effects of inflammation through the increased cytokine production (IL2) and in cell survival of CD4+ OX40+ T cells
T-cell receptor 4-1BB	Co-stimulatory receptor implicated in survival and expansion of activated T cells
Receptor activator of NF-κB-RANK	NFkB is a member of the tumor necrosis factor (TNF) receptor family. Implicated in osteoclast differentiation and in focal bone erosion and bone malignancies
CD28	Co-stimulatory receptor implicated in activation of T lymphocytes
**Surface transmembrane glycoproteins**	
CD133	Cancer stem cells marker
CD30 TNFRSF8	Hematological malignancies biomarker
**Others**	
Cytotoxic T cell antigen-4-CTLA-4	Receptor expressed on activated T cells. Reduction in T-cell responses and activation
B-cell–activating factor (BAFF)-receptor (BAFF-R)	BAFF is a member of the tumor necrosis factor (TNF) family cytokines. BAFF receptor is expressed on B-cells. Implicated in proliferation, maturation and cell survival of cancer B cells
CD124 (IL-4Rα)	Implicated in survival and in suppressor activity of myeloid derived suppressor cells
VCAM-1	Member of the immunoglobulin-like super family. Implicated in recruitment of immune cells to inflammation sites
Toll-like receptor 3 ectodomain	Implicated in the production of INF-β and inflammatory cytokine
hyaluronic acid (HA) binding domain of CD44	Belongs to the proteoglycan family of transmembrane glycoproteins. Receptor for hyaluronic acid. Role in tumor growth and metastasis. Marker for subpopulations of tumor initiating cells and implicated in chemo resistance
Angiopoietin-1	Promotes endothelial cell survival and vascular impermeability. Prevents increase in endothelial cell adhesion molecule expression
Angiopoietin-2	Potentialisation of proangiogenic growth factors
CD71	Responsible for cellular iron transport. Rapidly proliferative cells require more iron.
	Cofactor for enzyme involved in DNA synthesis of proliferative cells
Multidrug resistant-associated protein 1-MRP1	MRP1 correlates with chemotherapy drug resistance. Expressed in cancer stem cells
CD16α	Implicated in antibody dependent cellular cytotoxicity and recruitment of NK cells. NK cells are important for suppressing tumor metastasis and outgrowth
Alkaline phosphatase placental-like 2-ALPPL-2	Implicated in cell growth and invasion. Ectopically expressed on cell surface and in cell secretion
Nucleolin	Multifunctional protein interacting with DNA and RNA. Involved in rRNA maturation, ribosome assembly and exportation of ribosome components to the cytoplasm
	Role in mRNA stabilization (example: role in stabilization of bcl-2 mRNA in human leukemia). Overexpressed on the surface of certain cancer cells
Immunoglobulin heavy mu chain	Major component of the B-cell antigen receptor in Burkitt-s lymphoma cells
	Implicated in transforming healthy cells and in cancer progression

**Table 2:** Aptamers to tumor cell-surface biomarkers selected by protein-based SELEX (updated since 2011 [42,43,44]).

Biomarker	Target Used for Protein-SELEX	Aptamer Library	Number of Variable Nucleotides	Number of Rounds of Selection	Separation Method	Name of Selected Aptamers	K_D_	Applications	Year	References
**Cell-adhesion molecules**										
Epithelial cell adhesion molecule-EpCAM (CD326)	His-tagged C-terminal domain of EpCAM	2′F-RNA	40	12	affinity chromatography	Truncated EpCAM RNA aptamer	55 nM to human cells	Target stem cell marker. Potential applications: development of targeted cancer nanomedecine and molecular imaging agents	2011	[45]
EpCAM	His-tagged C-terminal domain of EpCAM	DNA	40	12	affinity chromatography	Truncated aptamer SYL3C	38 and 67 nM to cells	Imaging (confocal) Cancer cell capture	2013	[46]
Carcinoembryonic antigen- CEA (CD66e)	His-tagged recombinant protein of full-length CEA	2′F-RNA	40	17	affinity chromatography	Group I, II and III	low nM range	Inhibition of cell migration/invasion in vivo. Promotion of cell anoikis resistance in vitro Inhibition of liver metastasis in vivo	2012	[47]
Integrin αvβ3	purified αvβ3 integrin	2′F-RNA	50	15–17	affinity chromatography	Apt-avb3	nM range	Inhibition of endothelial cell adhesion and proliferation Reduction of endothelial cell tube formation Inhibition of cancer cell proliferation and adhesion (HUVEC) Increases endothelial cell apoptosis	2005	[48,49]
Integrin αvβ3	purified αvβ3 integrin	2′F-RNA	50	6	MAI-SELEX ^1^	αV-1 and β3-1	8.9–10.5 nM	Recognizes distinct binding sites on a single target ( αV or β3) with minimal cross-reactivity Potential applications: molecular diagnosis and targeted therapies	2012	[50]
E-and P-Selectin	recombinant human E-selectin/IgG-Fc-chimeras	DNA	50	17	affinity chromatography	SDA	100 nM	Inhibition of cancer cell adhesion Potential applications in therapies during metastasis formation	2014	[51]
L-Selectin	L-selectin-Ig chimera	2′-NH2 RNA	40	14	affinity chromatography	14.12	0.2–3 nM range	Preferential blokade of a specific selectin	1996	[52]
L-Selectin	L-selectin–IgG fusion protein	DNA	40	17	affinity chromatography	LD201, LD174 and LD196	1.8, 5.5 and 3.1 nM	Inhibition of lymphocyte rolling on endothelial cells	1996	[53]
**Tyrosine kinase receptors**										
EGFR	purified extracellular domain of human EGFR	RNA	62	12	not documented	J18	7 nM	Drug delivery (internalization of gold nanoparticules) Potential application: delivery of siRNA and cancer detection	2010	[54]
EGFR	human EGFR-Fc protein	2′F-RNA	62	9 + 7–9 rounds with a 30% doped sequence (from aptamer E01)	affinity chromatography	E07 Internalized	2.4 nM	Prevention of proliferation of tumor cells (blocks receptor autophosphorylation) Drug delivery (Gemcitabine) and induces cell death	2011	[55,56]
EGFR	purified extracellular domain of EGFR	DNA	40	11	affinity chromatography	Tutu-22	56 nM	Regognizes EGFR-positive cancer cells with strong affinity and selectivity Potential applications: development of novel targeted cancer detection, imaging and therapy	2014	[57]
EGFRVIII	histidine-tagged EGFRvIII ectodomain (*E. coli.* system)	2′F-RNA	40	12	affinity chromatography	E21	33 nM	Disruption of post-translational modifications of immature EGFRvIII Induction of apoptosis	2009	[58]
HER-2	recombinant glutathione S-transferase (GST)-tagged ErbB2 protein (22–122 amino acids)	2′F-RNA	50	15	affinity chromatography	SE15-8	low nM range	High specificity to ErbB2 and not to other members of the ErbB family Potential applications: drug delivery and imaging for in vivo diagnosis	2011	[59]
HER-2	peptide from the juxtamembrane region of HER2 extracellular domain	DNA	40	multiple	affinity chromatography	HB5	18.9 nM	Drug delivery (Doxorubicin)	2012	[60]
HER-2	20-amino acid HER2 peptide	Thio-DNA	21	12	affinity chromatography	HY6	172 nM	Potential application: targeted therapy	2015	[61]
HER-2	His-tagged Her2–extra cellular domain (*E. coli* system)	DNA	40	15	membrane filtration	ECD_Apt1	6.33 nM	Potential applications: theranostic (non invasive cancer diagnosis), therapeutics and monitoring patient compliance	2017	[62]
HER-3	extracellular domains of HER3 produced in S2 insect cells	RNA	49	15	membrane filtration and gel shift assay	A30	0.1 nM range	Inhibition of HER3 activation and growth of tumor cells Potential application: anticancer drug	2003	[63]
c-MET	c-Met-Fc	DNA	40	12	membrane filtration	CLN3 and CLN4	91 pm and 11 nM	Mediates tumor cell lysis Recruits NK cells to tumor and induces ADCC	2011	[64]
c-MET	c-Met-Fc	2′F-RNA	40	16	membrane filtration	CLN64	1 nM	Inhibition of tumor cell migration Potential application: therapeutics and diagnosis	2015	[65]
**Cell membrane-associated enzyme**										
Prostate specific membrane antigen-PSMA	706 extracellular amino acids of PMSA	2′F-RNA	40	6	affinity chromatography	A9, A10, A10-3 after minimization & optimization	low nM range	Promotion of tumor regression Delivery of siRNA Potential application: diagnosis and therapies	2002	[66,67]
**Mucins**										
MUC-1	MUC-1 peptides of 2 lenghts: 9 and 60 amino acids	DNA	25	10	affinity chromatography	S1.3/S2.2	low nM range to 0.1 nM	Potential application: detection by fluorescent microscopy	2006	[68]
MUC-1	His-tagged unglycosylated form of the MUC1 protein containing five tandem repeats of the VTR (*E. coli* system)	DNA	25	10	affinity chromatography	MUC1-5TR-1, 2, 3, 4	47–85 nM	Potential application: diagnosis assays for early or metastatic diseases	2008	[69]
**Tumor necrosis factor receptor (TNF-R) and co-stimulatory receptors**										
T-cell receptor OX40	extracellular domain of OX40-Fc fusion protein	2′F-RNA	40	9–11	affinity chromatography	9C7, 11F11	2-10 nM for purified OX40 protein and # 50 nM for OX40 on activated T cells	Increasing proliferation of T lymphocytes and production of IFN-γ. Potential application: therapeutics in association with dendritic cell-based vaccines (adoptive cellular therapy)	2013	[70]
T-cell receptor OX40	murine extracellular domain of OX40-Fc fusion protein	2′F-RNA	40	11	affinity chromatography	9.8	8 nM	Induces nuclear localization of NFκB, cytokine production and cell proliferation. Increases dendritic cell based tumor vaccine effects	2008	[71]
T-cell receptor 4-1BB	murine extracellular domain of 4-1BB-Fc fusion protein	2′F-RNA	40	12	affinity chromatography	M12-23 (multimeric aptamer)	40 nM	Inhibition of tumor growth in vivo. Potential application: therapeutic manipulation of the immune system	2008	[72]
Receptor activator of NF-κB-RANK	recombinant human soluble RANK/IgG_1_Fc chimera	RNA	40	7	affinity chromatography	apt1, apt2 and apt3	0.33, 1.8 and 5.8 μM. 100 nM for the 2′-F version of aptamers	Potential application: therapeutics against osteoclastogenesis	2004	[73]
CD28 ^2^	murine recombinant CD28-Fc fusion protein	2′F-RNA	25	9	affinity chromatography	CD28Apt2 and CD28Apt7	60 nM for CD28Apt7-dimer	Potentialisation of antitumor vaccine efficacy Reduction of tumor progression and increased overall survival (in vivo) Potential application: enhancing vaccine-induced immune responses	2013	[74]
**Others**										
Cytotoxic T cell Antigen-4-CTLA-4	murine CTLA-4/Fc fusion protein	2′F-RNA	40	9	membrane filtration	M9-9	30–60 nM	Increases tumor immunity (in vivo) Potential application: immunotherapy	2003	[75]
B-cell–activating factor (BAFF)-receptor (BAFF-R)	Human recombinant BAFF-R protein	2′F-RNA	50	12	membrane filtration	R-1, R-2 and R-14	47, 95 and 96 nM	Delivery of siRNA. Potential application: combinatorial therapeutics	2013	[76]
CD124 (IL-4Rα)	recombinant ILR4α protein enzymatically cleaved	2′F-RNA	40	5	affinity chromatography	cL42	14 nM for recombinant protein and 788 nM for MCS2 cells	Induction of MDSCS apoptosis Promotes CD8+ T cell infiltration and reduces the number of MDSCs infiltration. Reduction of tumor progression in vivo	2012	[77]
VCAM-1	N-terminal fragment of VCAM-1	2′F-RNA	40	12	affinity chromatography	12.11	10 nM	Potential application: imaging	2007	[78]
Toll-like receptor 3 ectodomain	Toll-like receptor 3 ectodomain with N-terminal FLAG and C-terminal His	RNA	40	7	membrane filtration	Family-I and Family II	# 3 nM	Aptamer without agonist and antagonist effects	2006	[79]
hyaluronic acid (HA) binding domain of CD44	HA-binding domain of human CD44 (cell-free expression system)	Thio-DNA	30	10	affinity chromatography	TA1-TA6	180–295 nM	Potential applications targeted therapy and imaging	2010	[80]
CD44	GST-tagged human recombinant full length CD44 protein	2′F-RNA	45	11	affinity chromatography	Apt1	81.3 nM	Potential applications therapeutic (targeted delivery againt stem cells) and diagnosis	2013	[81]
Angiopoietin-1	recombinant human Ang1	2′F-RNA	40	9	membrane filtration	ANG9-4	2.8 nM	Inhibition of cell endothelial cell survival	2008	[82]
Angiopoietin-2	recombinant human Ang2	2′F-RNA	40	11	membrane filtration	11-1 and truncated 11-1.41	3.1 and 2.2 nM	Inhibition of angiogeneis (in vivo)	2003	[83]

^1^ Integrin αvβ3 is a heterodimeric transmembrane protein composed of α and β chains, for which the selection procedure of a 2′-fluoro aptamer has been patented [48]. In order to select for aptamers specific to homodimer αv and β3, Gong et al [50], developed a strategy called MAI-SELEX (MAI for multivalent aptamer isolation). Two distinct selection stages were employed, the first being a classical affinity selection on the purified full-length αvβ3 integrin. The second module, for specificity, leads selection to β3 as integrin αIIbβ3 served as a protein decoy. Two aptamers, specific for αv and β3 were identified with affinities in the low nanomolar range. This selection strategy applied to heterodimeric proteins is limited to the availability of decoy proteins. ^2^ Aptamer, GR1, targets CD28. This G-rich oligonucleotide, which, alike AS1411 [84], has not selected by SELEX, inhibits CD28 T cell responses in vitro and in vivo [85].

**Table 3:** Aptamers selected by cell-based SELEX on pre-identified tumor cell-surface biomarkers.

Biomarker	Aptamer Library	Number of Variable Positions	K_D_ Range	Number of Positive Selection Rounds (*selection method*) ^1^	Cells Used for Positive Selection	Cells Used for Counter-SELEX	Applications	References
**Tyrosine kinase receptors**								
EGFRvIII	DNA	30	0.62–37.57 nM	14	U87-EGFRvIII, U87-MG, GBM cells overexpressing EGFRvIII	U87-MG	Potential applications: delivery of chemical drug and diagnosis	[117]
EGFRvIII	DNA	40	3–16 nM	11	U87-MG, GBM cells overexpressing EGFRvIII	U87-MG	Imaging (radiolabeled 188Re, in vivo) Potential applications: diagnosis in stratifying patient and monitoring treatment	[118]
HER-2	RNA	40	94.6 nM	20	HER-2-overexpressing SK-BR-3 cell line	MDA-MB-231, a HER-2-underexpressing breast cancer cell line	Potential application: therapy	[113]
HER-2	2′F-RNA	20	46–82 nM	9 (*cell-internalization SELEX*)	N202.1A mammary carcinoma clonal cell lines expressing high levels of surface HER-2/neu.	N202.1 E clonal cell line has no detectable surface expression of the HER2/neu oncoprotein.	Drug delivery (Bcl-2 siRNA) Induces chemosensibilisation and reduces drug resistance	[100]
HER-2	DNA	50	Not documented	4 rounds	Cleared extract of ErbB-2-overexpressing N87 cells	Not documented	Acceleration of ErbB-2 degradation in lysosomes Inhibition of growth of tumor cell in vitro and tumor mass in vivo	[116]
HER-2	DNA	30	5–23 nM	8 rounds of protein SELEX followed by 7 rounds of cell -SELEX (*hybrid-SELEX*)	HER-2 overexpressed in SKOV3 ovarian cancer cells	No cells	PET imaging (radio labeled, in vivo)	[109]
HER-2	DNA	40	Not documented	16	HER2- overexpressing breast cancer cell line, SK-BR3	HER2 negative breast cancer cell line, MDA-MB468	Development of a new method to monitor the enrichment of aptamers in a given round of cell-SELEX	[114]
Receptor tyrosine kinase-RET^C634Y^	2′F RNA	50	Tens of nM	15 rounds of selection on cells, followed by 7 rounds on purified protein (*hybrid-SELEX*)	RET^C634Y^ mutant receptor expressed in PC12 cells (PC12/MEN2A)	PC12	Potential applications: diagnosis, imaging and therapy	[108]
Neurotrophin receptor TrkB	2′F RNA	20	2 nM	4 (*cell-internalization SELEX*)	TrkB-expressing HEK cells	HEK	Neuroprotective effects Potential in therapy for neuro degenerative disease	[119]
Other kinase receptors								
TGFβ III receptor	2′F RNA	60	1 nM	11	TGFβ III receptor ectopically expressed on CHO-K1 cells	CHO-K1	Potential application: therapy through inhibition of TGFβIII receptor	[102]
Transferrin receptors (TfR)								
CD71	2′F-RNA	50	nM range	4 rounds of protein-SELEX on the His-tagged recombinant protein and 1 round on cells (*hybrid SELEX combined with cell-internalization SELEX*)	HeLa cells, a human cervical cancer cell line known to express TfR	NO cells	Delivery of siRNA Targets cell in liposomes	[94]
ATP-binding cassette (ABC) transporters								
Multidrug resistant-associated protein 1-MRP1	2′F-RNA	25	50 nM	10 rounds of peptide-SELEX followed by 1 round of cell-SELEX (*hybrid-SELEX*)	chemotherapy-resistant tumor cell line that has high MRP1 expression (H69AR)	Parental cell line H69	Reduction of cell growth in vitro and improved survival in vivo	[111]
**Cell adhesion molecules**								
Epithelial cell adhesion molecule-EpCAM (CD326)	DNA	40	μM range	7 (*FACS-SELEX*)	EpCAM over-expressed in HepG2 cells	HepG2 cells	Potential application: stem cell marker	[120]
Integrin α6β4	DNA	39	139 nM	5 rounds of cell-SELEX followed by 7 rounds of protein-SELEX (*hybrid-SELEX*)	PC-3 cells	PC-3 β4 integrin (ITGB4) knockdown cells	Imaging (confocal) Potential application: drug delivery	[110]
Integrin αv	RNA	35 & 40	359–408 nM	13 (*Isogenic cell-SELEX*)	Human HEK293 cells manipulated to generate positive αv selection cells by overexpressing ITGAV	Human HEK293 cells depleted in ITGAV with microRNA-mediated silencing.	Potential application: targeting channels, transporters…	[112]
Fc receptors								
CD16α	DNA	40	6–429 nM	9 rounds of protein-SELEX and 6 rounds of cell-SELEX (*hybrid-SELEX*)	CD16_-6His and CD16_ Val-158 or Phe-158 alloforms expressed on recombinant Jurkat cells	Jurkat E6.1 cell line	ADCC (tumor cell lysis)	[64]
Surface transmembrane glycoproteins								
CD133	2′F RNA	40	Not determined	6	HEK293T expressing CD133	His-tagged irrelevant protein expressed in HEK293T	Potential application: target cancer stem cells and molecular imaging	[121]
CD30 TNFRSF8	DNA	30	nM range	20 rounds of cell-SELEX followed by 5 rounds on purified His-tagged CD30 protein (*hybrid-SELEX*)	CD30-positive K299 T-cells lymphoma	Jurkat cells	Potential application: therapy	[115]

^1^ Selection method is specified if it is different from classical cell-SELEX.

**Table 4:** Aptamers Selected by Cell-Based SELEX on Post-Identified Tumor Cell-Surface Biomarkers.

Biomarker	Aptamer Library	Applications	References
EGFR	2′F-RNA	Induces EGFR-mediated signal pathways causing selective cell death Inhibits tumor growth in vivo Combined cetuximab-aptamer treatment shows clear synergy in inducing apoptosis in vitro and in vivo Potential application: translational therapy	[130]
PDGFR β	2′F-RNA	Inhibition of receptor signaling and of glioblastoma-derived tumor growth Inhibition of cell migration and proliferation Induction of differentiation	[99]
Alkaline phosphatase placental-like 2-ALPPL-2	2′F-RNA	Targets ALPPL-2 in both membrane bound and secretary forms Potential applications: diagnosis, imaging and therapy	[131]
selectin L and integrin α4	DNA	Potential application: therapeutic intervention	[132]
CD44/CD24	DNA	Potential applications: disruption of therapeutic resistance, invasion and angiogenesis	[133]
CD44	DNA	Potential applications: cancer detection, imaging and drug delivery	[134]
Protein Tyrosine Kinase 7-PTK7	DNA	Potential application: drug delivery	[135]
Insulin Receptor	2′-fluoro RNA	Inhibition of IR signaling Reduction of cell viability Potential applications: targeted therapies	[136]
Axl	2′F-RNA	Interferes with cell migration and invasion Inhibition of spheroid formation and cell transformation Inhibition of tumor growth	[137]
Nucleolin	DNA	Interferes with multiple biological activities in tumor cells Induction of apoptosis via down-regulation of bcl-2 proteins	[84,127,128,129]
Immunoglobulin heavy mu chain	DNA	Role in identification of cell membrane receptor with increased expression levels Potential applications: early diagnosis, targeted therapy and mechanistic studies	[138]
